# Area variations in multiple morbidity using a life table methodology

**DOI:** 10.1007/s10742-015-0142-4

**Published:** 2016-01-08

**Authors:** Peter Congdon

**Affiliations:** School of Geography and Life Sciences Institute, Queen Mary University of London, London, UK

**Keywords:** Multiple morbidity, Disease free life expectancy, Multinomial, Deprivation, Bayesian, Spatial

## Abstract

Analysis of healthy life expectancy is typically based on a binary distinction between health and ill-health. By contrast, this paper considers spatial modelling of disease free life expectancy taking account of the number of chronic conditions. Thus the analysis is based on population sub-groups with no disease, those with one disease only, and those with two or more diseases (multiple morbidity). Data on health status is accordingly modelled using a multinomial likelihood. The analysis uses data for 258 small areas in north London, and shows wide differences in the disease burden related to multiple morbidity. Strong associations between area socioeconomic deprivation and multiple morbidity are demonstrated, as well as strong spatial clustering.

## Background

A number of recent studies stress the health care implications of the increasing prevalence of long term chronic conditions, in particular people having two or more conditions (e.g. Bähler et al. 2015; Reeve et al. 2006; Mercer et al. 2009; Wolff et al. 2002; Diederichs et al. 2011). The coexistence of two or more conditions is known as multiple morbidity, complex morbidity, or as complex chronic disease (van Oostrom et al. 2014).

Long term chronic conditions are those for which there is currently no cure (Kings Fund 2015), and generally managed in primary care, for example: hypertension, diabetes, depression and chronic obstructive pulmonary disease. Such conditions are disproportionately concentrated among older persons (over 65) but also occur at significant levels among intermediate age groups such as 50–64-year-olds. Of relevance to health care management and resource allocation are the expected portions of lifetime spent with single and multiple conditions, and how far these differ by small area.

There is currently little evidence regarding socioeconomic differences in the burden of multiple long term conditions, such as average years lived with multiple chronic conditions as against years lived with a single condition. Formal statistical approaches to spatial analysis of healthy life expectancies (e.g. Jonker et al. 2013) are so far limited to treatments with health as a binary variable (with illness and health as the only states).

Multiple morbidity is an important influence on health care use (e.g. hospital admissions, average annual health care costs) and hence on differential health care burdens between population subgroups and different geographic areas (Wolff et al. 2002). For example, Payne et al. (2013) report physical multi-morbidity to be strongly associated with unplanned and preventable admissions to hospital, with risks of unplanned admission exacerbated by coexistent mental health conditions and socio-economic (area) deprivation. Such findings imply that area differences in the onset of multiple morbidity act as a central element in differential health needs and burdens between areas. There is also an increased recognition of multi-morbidity as a basis for population risk stratification, namely dividing populations into different risk strata with regard to predicting risks of specified outcomes such as unplanned admission to hospital (Paton et al. 2015).

Regarding impacts of geographic risk factors, there is evidence from the community health literature that the burden of multiple conditions is unequally distributed according to area socioeconomic status, and that the burden in deprived areas extends significantly into age groups under 65 (Barnett et al. 2012). However, such differentials have not so far been expressed in life year terms. Health and total expectancies in areas may also be affected by location of nursing homes (Nimmo et al. 2006), and by environmental factors such as greenspace (Jonker et al. 2014). However, formal evaluation of these effects and their relative importance has not so far been undertaken when the health outcomes include complex chronic disease.

The analysis here addresses these questions and is particularly oriented to small area comparisons in the context of growing multiple morbidity. It focuses on estimation of spatial life tables considering illness from a multinomial perspective, based on levels of treated prevalence of a range of chronic conditions. The three population health categories considered here are those without any condition, those with a single condition only, and those with multiple conditions (two or more). Life tables are then based on area data for mortality combined with multi-category morbidity data for areas.

As is generally the case for data with population coverage, transition data on moves between states are not available, and so the Sullivan method approximation to a multistate analysis is adopted (Lynch and Brown 2010), using a multinomial likelihood to reflect the three population health categories. An additional important feature of the analysis is that the focus is on period life and health expectancies (rather than cohort expectancies) (Office of National Statistics 2015a). Period expectancies at a given age for an area are the average number of years a person would live (or live without illness) if he or she experienced the particular area’s age-specific mortality or morbidity rates for that time period throughout their life. No allowance is made for projected changes in mortality or morbidity; also, people may live in other areas for at least some part of their lives.

Bayesian estimation via WINBUGS and Markov Chain Monte Carlo (MCMC) sampling (Lunn et al. 2009) is adopted as this provides stable estimation, based on borrowing strength over ages and areas (Jonker et al. 2013). Conventional life table methods use unsmoothed age–area specific mortality and illness rates (without any borrowing of strength), which for relatively small populations may show variance instability (Anselin et al. 2006), leading to wide confidence intervals for expectancy estimates.

A Bayesian approach also facilitates estimation of sampling properties (such as 95 % intervals) of complex summary indicators (such as life expectancies and spatial correlation indices). A Bayesian analysis provides a natural framework for modelling spatially clustered area random effects, reflecting unmeasured area risk factors.

## Case study

The analysis here focuses on single condition and multiple condition morbidity based on patient register data for diagnosed conditions treated in primary care by the National Health Service (NHS). The analysis is according to patient place of residence in one of 258 small areas in two London boroughs (Barking and Dagenham, Havering) for the illness data; and by place of residence at death, for the mortality data.

Multiple morbidity is defined as the presence of two or more of 12 conditions in the year 2011: coronary heart disease, heart failure, hypertension, stroke, diabetes, asthma, chronic obstructive pulmonary disease, dementia, depression, serious mental illness (psychosis or bipolar disorder), cancer, and chronic kidney disease. This range of conditions is similar to that in the studies considered by Diederichs et al. (2011). Deaths data are for the 5-year period 2009–2013.

With regard to differences in health care usage according to levels of morbidity, Table 1 distinguishes patients in the study region according to age group, number of long term conditions (0, 1, 2 or more), and selected health outcomes in 2011–2012. These are unplanned (emergency) admissions and inpatient bed days. It can be seen that having two or more long term conditions is associated with much enhanced levels of unplanned admission and inpatient bed days, as compared to those with no conditions or only one condition. The excess in use is particularly apparent for patients under 65, and in early old age, 65–74. The latter population is important as it is relatively large in numerical terms compared to populations over 75 (and so can potentially generate more health care events), and the analysis below shows expected life spans before onset of multiple morbidity are generally in this range; that is, multiple morbidity typically commences between ages 65 and 74.

**Table 1:** Annual average inpatient usage by age group and morbidity category (number of long term conditions)

Number of conditions	Under 65	65–74	75 plus	All ages
Unplanned (emergency) admissions				
No condition	0.02	0.03	0.08	0.03
One condition only	0.06	0.05	0.12	0.06
Two or more conditions	0.14	0.14	0.25	0.18
Inpatient bed days (all admission types)				
No condition	0.15	0.27	0.99	0.17
One condition only	0.32	0.54	1.43	0.46
Two or more conditions	0.92	1.47	2.69	1.70

Study region 2011–2012

The area framework is defined by lower level super output areas (LSOAs), which are Census based small areas with an average of 1500 residents and 650 households, and with just under 35,000 LSOAs across England. LSOAs are derived from smaller Census output areas, subject to constraints of proximity (to ensure a compact shape), and social homogeneity within each LSOA. Specifically, homogeneity is based on dwelling type (e.g. detached/semi-detached, etc.) and nature of tenure (e.g. owner-occupied, private rented, etc.) (Office of National Statistics 2015b). The importance of housing context in health outcomes is attested in a number of studies (Dunn and Hayes 2000; Macintyre et al. 2003). For ease of reference, LSOAs are referred to subsequently as neighbourhoods.

Residential stability within such neighbourhoods is relatively high (as compared to, say, more transient inner city areas). Population turnover, especially among older people where morbidity and mortality rates are highest, is relatively low. For example, data from the NHS Central Register (Office of National Statistics 2015c) for migrant flows by people over 65 show 850 immigrants and 1050 emigrants for the entire study region in the year to June 2010, as compared to a population of 61,600 aged over 65 (2011 Census).

The study region shows wide differences in socio-economic conditions. Ten of the 258 neighbourhoods in the case study region are in the most affluent decile regarded from a national (England-wide) perspective: that is, these neighbourhoods are among the most affluent 10 % of the 32,482 LSOAs across England. At the other extreme, 13 of the 258 neighbourhoods are among the most deprived 10 % of all English LSOA.

Average rates of multiple morbidity in the study region are strongly age related: percentages among people aged over 75 are 52 and 59 % among females and males, respectively, approximately double the rate among those aged 60–74, namely 25 (females) and 30 % (males).

## Methods

For notational convenience consider deaths, health and population data for a particular gender. Let A and X denote the number of areas and age bands. Deaths are generally obtained over a multiyear period, whereas prevalence data are for a single year.

Regarding the population denominator for deaths, let T_ax_ denote population years for area a (a = 1, …, A), and age x (=1, …, X), over a multiyear period, and D_ax_ denote deaths over that period. Then deaths are assumed binomial with unknown death rates ρ_ax_, namely

1 $${\text{D}}_{\text{ax}} \sim{\text{Binomial}}({\text{T}}_{\text{ax}} ,\uprho_{\text{ax}} ).$$

For modelling death rates ρ_ax_ we assume a regression on age, area and age–area interactions, as well as impacts of known area risk factors.

Age effects are taken to be random effects represented using a first order random walk

2 $${\text{b}}_{\text{x}} \sim{\text{N}}({\text{b}}_{{{\text{x}} - 1}} ,\upxi)$$

where ξ is a variance, and the initial age effect is assigned a diffuse normal prior.

Area effects r_a_ are modelled using a spatially autoregressive prior (Besag et al. 1991). Let C = [c_ab_] denote a symmetric spatial interaction matrix between areas a and b, then the conditional prior for r_a_ conditioning on effects r_[a]_ in remaining areas b ≠ a is

3 $${\text{r}}_{\text{a}} |{\text{r}}_{{\left[ {\text{a}} \right]}} \sim{\rm N}(\upomega_{\text{a}} ,\uptau_{\text{a}} ),$$

where ω_a_ = Σ_b_c_ab_r_b_/Σ_b_c_ab_ is a weighted average of surrounding neighbourhood effects, and τ_a_ = κ/Σ_b_c_ab_ is a variance parameter. If the c_ab_ are binary, and based on whether areas a and b are adjacent or not, then Σ_b_c_ab_ is the number of areas contiguous to area a.

Area–age interactions are also likely: for example, mortality at middle ages may be higher in some areas. Such interactions, u_ax_, are assumed Normal with age specific variances,

4 $${\text{u}}_{\text{ax}} \sim{\text{N}}\left( {0,\upphi_{\text{x}}^{{({\text{u}})}} } \right).$$

To assess the need for interaction effects, a spike–slab prior is adopted within age bands, so that

5 $${\text{u}}_{\text{ax}} \sim\Omega _{\text{x}}^{{({\text{u}})}} {\text{N}}\left( {0, \,\upphi_{\text{x}}^{{({\text{u}})}} } \right) + \left( {1 - \,\Omega _{\text{x}}^{{({\text{u}})}} } \right)\Delta _{0}$$

where Δ_0_ denotes a unit point measure concentrated at zero, $\Omega _{\text{x}}^{{ ( {\text{u}})}} \sim {\text{Bern(}}\Upsilon _{\text{x}}^{{ ( {\text{u}})}} )$ are binary, and the retention probabilities $\Upsilon _{\text{x}}^{{ ( {\text{u}})}}$ may be preset (e.g. $\Upsilon _{\text{x}}^{{ ( {\text{u}})}} = 0.5$ or 0.1) or assigned a beta prior. So if $\Omega _{\text{x}}^{{({\text{u}})}} = 0$, the interaction terms for age group x are not included.

Mortality is also likely to be affected by known area risk factors X_a_ (e.g. socioeconomic deprivation influences premature mortality). Then with γ denoting an intercept, a logit link regression specifies

6 $${\text{logit}}(\uprho_{\text{ax}} ) =\upgamma + {\text{b}}_{\text{x}} + {\text{r}}_{\text{a}} + {\text{u}}_{\text{ax}} + {\text{X}}_{\text{a}}\upalpha,$$

where α denotes regression parameters.

Health data refer to prevalent cases in a particular year. Let H_ax_ = (H_ax0_, H_ax1_, H_ax2_) denote population totals disaggregated by area a, age band x, and health status category: 0 (no long term chronic conditions), 1 (one condition only) and 2 (two or more conditions). Health category data are assumed multinomial in relation to annual population totals P_ax_, namely

7 $${\text{H}}_{\text{ax}} \sim{\text{Multinomial}}({\text{P}}_{\text{ax}} ,\uppi_{\text{ax}} )$$

where π_ax_ = (π_ax0_, π_ax1_, π_ax2_).

We assume health status category 0 (no long term conditions) is the reference category, with the multinomial probabilities then obtained as follows:

8 $$\uppi_{\text{axj}} = {{\exp (\upeta_{\text{axj}} )} \mathord{\left/ {\vphantom {{\exp (\upeta_{\text{axj}} )} {\sum\limits_{{{\text{k}} = 0}}^{2} {\exp (\upeta_{\text{axk}} )} }}} \right. \kern-0pt} {\sum\limits_{{{\text{k}} = 0}}^{2} {\exp (\upeta_{\text{axk}} )} }}$$

9 $$\upeta_{\text{ax0}} = 0$$

10 $$\upeta_{\text{axk}} =\updelta_{\text{k}} + {\text{c}}_{\text{xk}} + {\text{s}}_{\text{ak}} +\upnu_{\text{axk}} + {\text{X}}_{\text{a}}\upbeta_{\text{k}} ,\quad {\text{k}} = 1,2.$$

The regression terms parallel those for mortality. Thus δ_k_ are intercepts, and c_xk_ ~ N(c_x−1,k_, χ_k_) denote age effects on single and multiple morbidity, which are again first order random walks with variances χ_k_. The s_ak_ are conditional autoregressive effects, as in (3), over areas a = 1, …, A, but specific to morbidity category k. The β_k_ are regression coefficients for known area risk factors.

The ν_axk_ are Normally distributed age–area interactions, as in (4), but specific to morbidity category k. These are subject to retention or exclusion within age bands via a spike–slab prior, as in (5). Thus

11 $$\upnu_{\text{axk}} \sim\Omega _{\text{x}}^{{\upnu ( {\text{k)}}}} {\text{N}}(0,\upphi_{\text{x}}^{{\upnu ( {\text{k)}}}} ) + \left( {1 -\Omega _{\text{x}}^{{\upnu ( {\text{k)}}}} } \right)\Delta _{0} ,$$

where $$\Omega _{\text{x}}^{{\upnu ( {\text{k)}}}} \sim{\text{Bern(}}\Upsilon _{\text{x}}^{{\upnu ( {\text{k)}}}} )$$.

To obtain life table summary statistics, assume equal length age intervals n_x_ = n with average fraction 0.5 of each interval survived. Then life table death probabilities _n_q_ax_ are obtained from area–age specific mortality rates which here are modelled rates ρ_ax_. Then

12 $$_{\text{n}} {\text{q}}_{\text{ax}} =\uprho_{\text{ax}} /\left( {1 + 0.5\uprho_{\text{ax}} } \right)$$

From the _n_q_ax_ are obtained survivor and years-lived functions, denoted *ℓ*_ax_ and L_ax_, and average life spans E_ax_ at exact age x. Disease free life expectancies HLE_ax1_ (free of a single chronic condition) and HLE_ax2_ (free of multiple conditions) are obtained using a multinomial extension of Sullivan’s method (Romero et al. 2005), namely

13 $${\text{HLE}}_{\text{ax1}} = \tfrac{1}{{\ell_{\text{ax}} }}\sum\limits_{{{\text{x}} = 1}}^{\text{X}} {\uppi_{{{\text{ax}}0}} {\text{L}}_{\text{ax}} } = \tfrac{1}{{\ell_{\text{ax}} }}\sum\limits_{{{\text{x}} = 1}}^{\text{X}} {(1 -\uppi_{{{\text{ax}}1}} -\uppi_{{{\text{ax}}2}} ){\text{L}}_{\text{ax}} } ,$$

14 $${\text{HLE}}_{{{\text{ax}}2}} = \tfrac{1}{{\ell_{\text{ax}} }}\sum\limits_{{{\text{x}} = 1}}^{\text{X}} {(\uppi_{{{\text{ax}}0}} +\uppi_{{{\text{ax}}1}} ){\text{L}}_{\text{ax}} } = \tfrac{1}{{\ell_{\text{ax}} }}\sum\limits_{{{\text{x}} = 1}}^{\text{X}} {(1 -\uppi_{{{\text{ax}}2}} ){\text{L}}_{\text{ax}} } .$$

Typically one is interested in variation between areas a = 1, …, A in total and healthy life expectancies at birth, denoted E_a_ = E_a0_, HLE_a1_ = HLE_a01_, and HLE_a2_ = HLE_a02_. Other age points may be of epidemiological concern (WHO 1984, p. 27).

As well as spatial variability in expected lifespans before multiple morbidity, one may be interested in ratio comparisons, such as expected lifetime spent with multiple chronic disease as compared with expected lifetime spent with a single condition. This can be measured by the ratios

15 $${\text{M}}_{{ 1 {\text{a}}}} = \left( {{\text{E}}_{\text{a}} - {\text{HLE}}_{{{\text{a}}2}} } \right) /\left( {{\text{HLE}}_{{{\text{a}}2}} - {\text{HLE}}_{{{\text{a}}1}} } \right),$$

which are termed complex morbidity ratios. One may also be interested in the expected proportions M_2a_ spent in different morbidity categories according to age, such as the expected proportion of ages 65–74 spent in multiple morbidity. One can estimate these proportions at area level by monitoring the indicators

16 $${\text{I}}(65 > {\text{HLE}}_{{{\text{a}}2}} ) + {\text{I}}(75 > {\text{HLE}}_{{{\text{a}}2}} ){\text{I}}({\text{HLE}}_{{{\text{a}}2}} \ge 65)(75 - {\text{HLE}}_{{{\text{a}}2}} )/10,$$

where I(A) = 1 if condition A is true, and I(A) = 0 otherwise. So for an area with HLE_a2_ under 65 (expected lifetime before onset of multiple morbidity is under 65), one has M_2a_ = 1, while for HLE_a2_ = 67 (say) one would have M_2a_ = 0.8. These proportions are stochastic and can be estimated by monitoring over MCMC iterations.

## Analysis

The life table analysis uses X = 18 quinquennial age bands (0–4, 5–9, through to 75–79, 80–84, and 85 plus), with separate analysis of males and females undertaken. There are A = 258 areas, with binary spatial interactions C = [c_ab_] based on whether areas a and b are adjacent or not.

Regarding known risk factors X_a_ with a potential impact on mortality, we consider socioeconomic deprivation, X_a1_; whether the LSOA area contains a nursing home, X_a2_; and the percent of the area consisting of greenspace, X_a3_. Socioeconomic economic deprivation is measured by the index of multiple deprivation or IMD (DCLG 2011). Covariates are standardised so their relative importance can be assessed within each outcome.

As mentioned above, area deprivation effects on mortality and ill-health are well established, though there is little evidence relating to area deprivation and multiple morbidity. Since deprivation effects may be stronger for younger subjects (Barnett et al. 2012, p. 39; Romeri et al. 2006, p. 22), we allow the effect of deprivation in Eqs. (6) and (10) to differ according to ages under and over 65. Thus age is an effect modifier for area deprivation. A form of effect modification applies to the nursing home effect, since it is confined to ages over 80, as most frail elderly are over 80.

For the MCMC analysis, we assume gamma priors with shape 1 and index 0.01 on inverse variance parameters, and Normal priors with mean zero and precision 0.001 on fixed effects (such as intercepts and regression coefficients). Beta(1, 1) priors are adopted on the interaction retention probabilities in (5) and (11). Estimates are based on the second halves of two chain runs of 10,000 iterations, with convergence assessed using Brooks–Gelman–Rubin diagnostics (Brooks and Gelman 1998).

Let Y = (D, H) denote the observations on death and disease. Posterior predictive checks are applied, based on predictions Y_new,ax_ sampled from the posterior predictive density. Firstly, we consider consonance between data and predictions by obtaining percentages of observations actually falling outside 95 % predictive intervals, namely the 95 % intervals of Y_new_ (Gelfand 1996). For a satisfactory model, one would expect the proportions of Y_ax_ falling outside the predictive intervals to be 5 % or less.

One may also consider predictive checks using summary fit measures. With F and F_new_ denoting fit measures using Y and Y_new_ respectively, posterior predictive *p* values are estimated by the proportion of iterations where F_new_ > F, with extreme *p* values (under 0.05 or over 0.95) indicating model discrepancies (Berkhof et al. 2000). Here Chi square fit is used, so for the binomial deaths data, F = ∑_ax_(D_ax_ − T_ax_ρ_ax_)^2^/[T_ax_ρ_ax_(1 − ρ_ax_)], and F_new_ = ∑_ax_(D_new,ax_ − T_ax_ρ_ax_)^2^/[T_ax_ρ_ax_(1 − ρ_ax_)]. For the multinomial health data with categories k = 0, 1, 2 (well, one condition, two or more conditions) the fit measures are F = ∑_axk_(H_axk_ − P_ax_π_axk_)^2^/[P_ax_π_axk_] and F_new_ = ∑_axk_(H_new,axk_ − P_ax_π_axk_)^2^/[P_ax_π_axk_].

## Results

Table 2 shows satisfactory posterior predictive checks: replicates sampled from the death and health data models are consistent with the observations. Table 3 shows estimated regression coefficients, by gender, for deprivation, nursing home location and greenspace. It can be seen that deprivation effects are strongest for ages under 65, and for multiple morbidity. Additionally deprivation effects on multiple morbidity are stronger for females than males. Nursing home location is a significant influence on mortality, especially for females, but not morbidity. Greenspace effects are not significant. Deprivation effects are thus considerably more pronounced than impacts of other area variables.

**Table 2:** Posterior predictive checks

Observations	Males	Females
Chi square posterior predictive probability		
Deaths	0.32	0.14
Wellness	0.15	0.44
Percentage of observations outside 95 % predictive interval		
Deaths	2.0	2.2
One condition	1.4	1.0
Two or more conditions	1.2	1.4

**Table 3:** Regression parameters

Risk factor	Modifier	Outcome	Posterior mean	2.5 %	97.5 %
*Males*					
Deprivation	Ages over 65	Mortality	0.139	0.080	0.196
		First condition	0.035	−0.012	0.084
		Multiple conditions	0.063	0.010	0.121
	Ages under 65	Mortality	0.246	0.176	0.313
		First condition	0.013	−0.024	0.049
		Multiple conditions	0.156	0.102	0.212
Nursing home		Mortality	0.078	0.032	0.125
		First condition	0.008	−0.073	0.078
		Multiple conditions	0.031	−0.030	0.095
Greenspace		Mortality	0.017	−0.018	0.052
		First condition	−0.008	−0.030	0.012
		Multiple conditions	−0.012	−0.040	0.016
*Females*					
Deprivation	Ages over 65	Mortality	0.135	0.068	0.200
		First condition	0.091	0.044	0.135
		Multiple conditions	0.165	0.110	0.222
	Ages under 65	Mortality	0.205	0.117	0.292
		First condition	0.051	0.019	0.084
		Multiple conditions	0.287	0.235	0.342
Nursing home		Mortality	0.108	0.062	0.155
		First condition	−0.035	−0.080	0.014
		Multiple conditions	−0.005	−0.051	0.042
Greenspace		Mortality	0.021	−0.019	0.062
		First condition	−0.011	−0.034	0.012
		Multiple conditions	−0.006	−0.037	0.023

Age–area interactions in the regression for single condition morbidity are retained across both genders for all ages, with posterior probabilities ${\text{Pr(}}\Omega _{\text{x}}^{{(\upnu1)}} )= 1|{\text{Y}}$) all exceeding 0.95. For mortality, such interactions are retained for males in age bands 65–69 and above, and for females in age bands 70–74 and above. For multi-morbidity among males, age–area interactions are retained for ages 10–24 and for ages above 35. For multi-morbidity among females, age–area interactions are retained for ages above 20.

### Deprivation gradients

The impact of area deprivation is also apparent in gradients in E_a_, HLE_a2_, M_1a_ and M_2a_ (for ages 65–74) when areas are arranged in ten decile groups, within the study region, according to their IMD score (Table 4). In particular, expected male lifetime HLE_a2_ without multiple morbidity in the most affluent areas stands at 71.8 compared to 66.0 in the most deprived areas, with the female contrast being 76.3 (most affluent areas) as compared to 69.5 (most deprived). The ratios M_1a_ (years with multiple morbidity as against years with a single condition) are highest for deprived areas, with the gradient being more pronounced for females.

**Table 4:** Contrasts in total life expectancy, healthy life expectancies, and morbidity ratios

	Total life expectancy		Expectancy before single condition		Expectancy before multiple morbidity		Complex morbidity ratio (M_1a_)		Proportion of age 65–74 in multiple morbidity (M_2a_)	
	Mean	95 % Interval	Mean	95 % Interval	Mean	95 % Interval	Mean	95 % Interval	Mean	95 % Interval
*Males*										
Decile 1	82.9	(81.9, 83.8)	56.7	(56.2, 57.1)	71.8	(71.2, 72.4)	0.73	(0.68, 0.78)	0.33	(0.27, 0.39)
Decile 2	81.7	(80.9, 82.6)	56	(55.6, 56.5)	71	(70.4, 71.6)	0.72	(0.68, 0.77)	0.40	(0.35, 0.45)
Decile 3	81.4	(80.6, 82.3)	55.8	(55.3, 56.2)	70.8	(70.2, 71.4)	0.72	(0.67, 0.76)	0.43	(0.37, 0.48)
Decile 4	80.4	(79.6, 81.2)	55.2	(54.8, 55.7)	70	(69.4, 70.6)	0.71	(0.67, 0.76)	0.48	(0.43, 0.54)
Decile 5	79.6	(78.7, 80.4)	54.8	(54.4, 55.2)	69.3	(68.7, 69.8)	0.72	(0.67, 0.77)	0.58	(0.53, 0.63)
Decile 6	79.1	(78.2, 79.9)	54.3	(53.9, 54.7)	68.8	(68.2, 69.3)	0.73	(0.68, 0.77)	0.64	(0.59, 0.69)
Decile 7	77.8	(76.9, 78.7)	54	(53.6, 54.4)	67.4	(66.8, 68)	0.79	(0.74, 0.85)	0.76	(0.7, 0.81)
Decile 8	77.3	(76.4, 78.2)	53.2	(52.8, 53.7)	66.7	(66.1, 67.3)	0.8	(0.75, 0.86)	0.83	(0.78, 0.88)
Decile 9	77.4	(76.4, 78.3)	53.4	(52.9, 53.8)	66.5	(65.9, 67.1)	0.84	(0.78, 0.9)	0.81	(0.76, 0.87)
Decile 10	76.1	(75.1, 77.1)	53	(52.5, 53.6)	66	(65.4, 66.7)	0.78	(0.72, 0.84)	0.87	(0.82, 0.92)
*Females*										
Decile 1	86.2	(85.1, 87.4)	60.2	(59.7, 60.7)	76.3	(75.5, 77)	0.62	(0.58, 0.67)	0.04	(0.02, 0.06)
Decile 2	86.6	(85.4, 87.7)	59.6	(59.2, 60.1)	75.8	(75.1, 76.5)	0.67	(0.62, 0.72)	0.05	(0.02, 0.08)
Decile 3	86.2	(85.1, 87.3)	59	(58.5, 59.5)	75.7	(75, 76.4)	0.63	(0.59, 0.67)	0.05	(0.03, 0.07)
Decile 4	86.4	(85.3, 87.7)	58.2	(57.7, 58.7)	74.8	(74, 75.6)	0.7	(0.66, 0.75)	0.11	(0.08, 0.16)
Decile 5	85.1	(84, 86.3)	57.5	(57, 58)	74	(73.3, 74.8)	0.67	(0.63, 0.72)	0.14	(0.1, 0.19)
Decile 6	83	(82.1, 84)	55.9	(55.5, 56.4)	71.9	(71.3, 72.6)	0.7	(0.65, 0.74)	0.32	(0.26, 0.38)
Decile 7	82.9	(81.8, 84)	55.3	(54.8, 55.7)	70.8	(70.2, 71.5)	0.78	(0.73, 0.84)	0.42	(0.36, 0.49)
Decile 8	81.3	(80.3, 82.3)	54.3	(53.8, 54.7)	69.7	(69, 70.3)	0.76	(0.71, 0.81)	0.54	(0.48, 0.6)
Decile 9	81.3	(80.3, 82.4)	54.3	(53.8, 54.7)	69.5	(68.8, 70.1)	0.79	(0.74, 0.85)	0.55	(0.48, 0.61)
Decile 10	81.4	(80.3, 82.6)	54	(53.4, 54.5)	69.5	(68.7, 70.2)	0.78	(0.72, 0.84)	0.55	(0.48, 0.61)

Decile 10 (most deprived), decile 1 (least deprived)

Posterior means and 95 % credible intervals

**Fig. 1:**
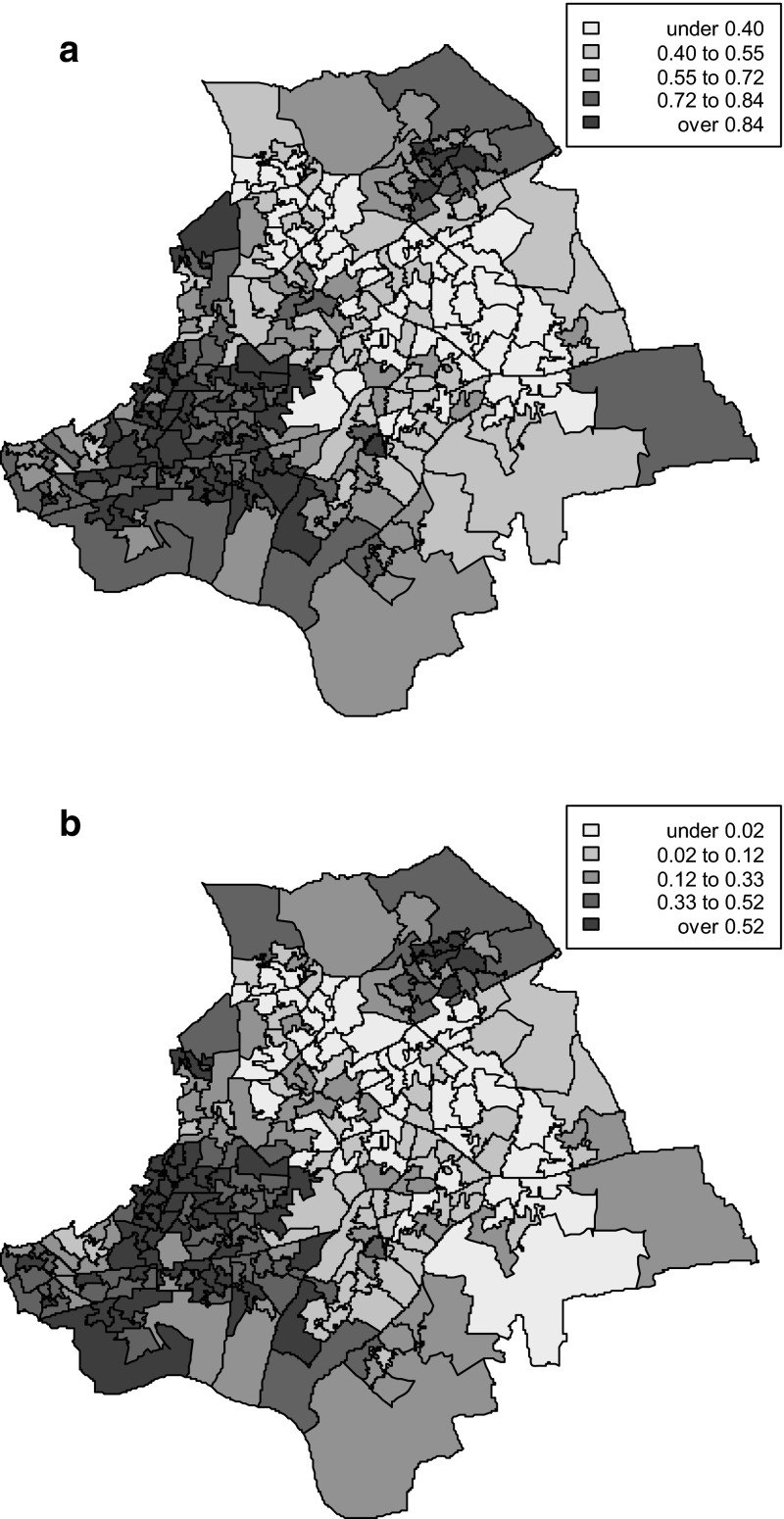
**a** Proportions of age band 65–74 in multiple morbidity, by small area (males). **b** Proportions of age band 65–74 in multiple morbidity, by small area (females)

Similarly, the proportions M_2a_ of early old age (the age band 65–74) spent in multiple morbidity are highest in deprived areas (last two columns, Table 4). Fig. 1a, b (with quantile cut-points) map out these proportions, and clear geographic differences can be seen. By virtue of the comparisons in usage shown in Table 1, proportions of early old age spent in multiple morbidity will translate into considerable differences in health care usage. This illustrates how multiple morbidity can be seen as mediating the effect of deprivation on health care use.

**Fig. 2:**
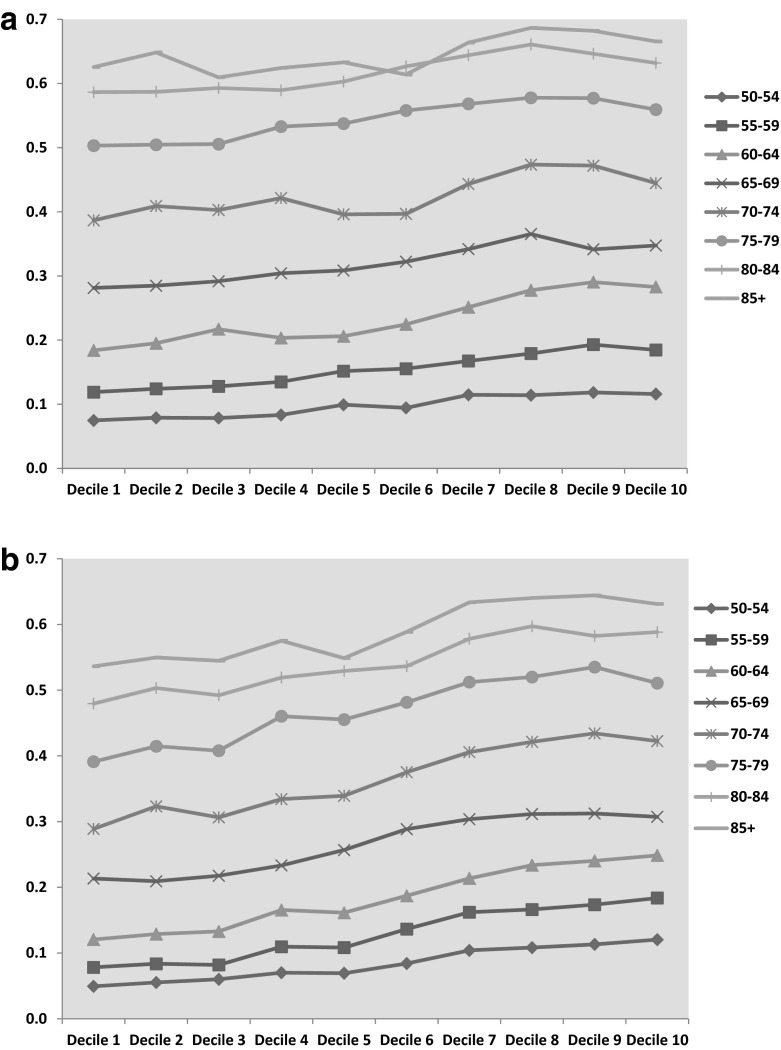
**a** Modelled rates of multiple morbidity, by age band and deprivation decile (males). **b** Modelled rates of multiple morbidity, by age band and deprivation decile (females)

Contrast in age-specific rates of multiple morbidity π_ax2_ also show a deprivation gradient, with this gradient tapering off among the very old (cf. Barnett et al. 2012). Table 5 and Fig. 2a, b, consider such rates for ages 50–54 and over. They show that the widest relative contrasts in rates between least and most affluent areas are at ages under 60.

**Table 5:** Contrasts in age-specific rates of multiple morbidity, by area deprivation decile

	Age group							
	50–54	55–59	60–64	65–69	70–74	75–79	80–84	85+
*Males*								
Decile 1	0.075	0.119	0.184	0.281	0.387	0.503	0.586	0.626
Decile 2	0.079	0.124	0.195	0.285	0.409	0.505	0.587	0.648
Decile 3	0.078	0.128	0.217	0.292	0.403	0.506	0.593	0.610
Decile 4	0.083	0.135	0.203	0.304	0.421	0.533	0.590	0.624
Decile 5	0.099	0.152	0.206	0.309	0.396	0.537	0.603	0.633
Decile 6	0.094	0.155	0.225	0.322	0.397	0.558	0.627	0.614
Decile 7	0.115	0.168	0.251	0.342	0.443	0.568	0.644	0.664
Decile 8	0.114	0.179	0.278	0.365	0.474	0.578	0.661	0.687
Decile 9	0.118	0.193	0.290	0.342	0.472	0.577	0.646	0.682
Decile 10	0.116	0.185	0.283	0.348	0.445	0.559	0.632	0.666
Ratio decile 10 to decile 1	1.55	1.55	1.54	1.24	1.15	1.11	1.08	1.06
*Females*								
Decile 1	0.049	0.078	0.121	0.213	0.289	0.391	0.480	0.536
Decile 2	0.055	0.084	0.129	0.209	0.323	0.415	0.503	0.550
Decile 3	0.060	0.082	0.133	0.218	0.306	0.408	0.492	0.545
Decile 4	0.070	0.110	0.166	0.233	0.334	0.460	0.519	0.575
Decile 5	0.069	0.108	0.161	0.257	0.339	0.455	0.529	0.549
Decile 6	0.084	0.137	0.187	0.289	0.375	0.482	0.537	0.589
Decile 7	0.104	0.162	0.214	0.304	0.406	0.512	0.578	0.634
Decile 8	0.108	0.166	0.234	0.312	0.421	0.520	0.597	0.640
Decile 9	0.113	0.174	0.240	0.312	0.434	0.535	0.583	0.644
Decile 10	0.120	0.184	0.249	0.307	0.423	0.511	0.588	0.631
Ratio decile 10 to decile 1	2.44	2.35	2.06	1.44	1.46	1.31	1.23	1.18

Decile 10 (most deprived), decile 1 (least deprived)

Posterior means and 95 % credible intervals

### Area differences and spatial clustering

As a final major aspect of socioeconomic contrasts in multi-morbidity, we consider directly age standardised area rates (Romeri et al. 2006). These are obtained by applying the 2013 European standard weights w_x_ (x = 1, …,X) to age-specific mortality and multiple morbidity rates. For example, the modelled area rates for multiple mortality over all ages are obtained as

17 $${\text{R}}_{\text{a}} = \sum\limits_{\text{x}} {{\text{w}}_{\text{x}}\uppi_{{{\text{ax}}2}} } .$$

For a restricted age range, x = x_1_ to x = x_2_, such rates are obtained as

18 $${\text{R}}_{\text{a}} = {{\sum\limits_{{{\text{x}} = {\text{x}}_{1} }}^{{{\text{x}}_{2} }} {{\text{w}}_{\text{x}}\uppi_{{{\text{ax}}2}} } } \mathord{\left/ {\vphantom {{\sum\limits_{{{\text{x}} = {\text{x}}_{1} }}^{{{\text{x}}_{2} }} {{\text{w}}_{\text{x}}\uppi_{{{\text{ax}}2}} } } {\sum\limits_{{{\text{x}} = {\text{x}}_{ 1} }}^{{{\text{x}}_{2} }} {{\text{w}}_{\text{x}} } }}} \right. \kern-0pt} {\sum\limits_{{{\text{x}} = {\text{x}}_{ 1} }}^{{{\text{x}}_{2} }} {{\text{w}}_{\text{x}} } }}.$$

Table 6 summarises area contrasts in all age mortality and multiple morbidity. Higher rates for both outcomes occur among males, with multi-morbidity among males of 13 % compared to 11 % among females (cf. Rizza et al. 2012). However, gender-specific contrasts between socio-economic extremes differ by outcome. The contrast in mortality rates (comparing decile 10 to decile 1) is greater for males, namely 60 % higher in the most deprived neighbourhoods as compared to the least deprived (cf. Romeri et al. 2006, p. 22).

**Table 6:** Neighbourhood mortality and multiple morbidity, age standardised rates per 1000, by area deprivation decile

	Mortality rate (per 1000)		Multi-morbidity rate (per 1000)	
	Mean	95 % Interval	Mean	95 % Interval
*Males*				
Decile 1	9.1	(8.5, 9.8)	114.4	(110, 118.9)
Decile 2	9.9	(9.2, 10.6)	118.1	(113.9, 122.5)
Decile 3	10.2	(9.5, 10.9)	118.8	(114.5, 123.2)
Decile 4	11.2	(10.5, 12)	121.8	(117.3, 126.5)
Decile 5	11.7	(10.9, 12.5)	124.7	(120.1, 129.3)
Decile 6	12	(11.2, 12.8)	128.5	(123.9, 133.3)
Decile 7	13.5	(12.6, 14.5)	138.6	(133.6, 143.7)
Decile 8	13.7	(12.7, 14.8)	144.8	(139.5, 150.2)
Decile 9	13.5	(12.5, 14.6)	145.9	(140.6, 151.3)
Decile 10	14.5	(13.4, 15.7)	142.5	(136.6, 148.4)
*Females*				
Decile 1	7.3	(6.7, 7.8)	86.7	(82.7, 90.8)
Decile 2	7.1	(6.6, 7.6)	91.9	(88, 95.9)
Decile 3	7.1	(6.6, 7.6)	91.6	(87.7, 95.6)
Decile 4	7.4	(6.9, 8)	102.7	(98.3, 107.3)
Decile 5	7.7	(7.1, 8.3)	103.4	(99.2, 107.9)
Decile 6	9.2	(8.6, 9.9)	115.3	(110.7, 120.1)
Decile 7	9.2	(8.5, 10)	126.7	(121.6, 132)
Decile 8	10.3	(9.5, 11.1)	131.5	(126.3, 137.2)
Decile 9	10.2	(9.4, 11)	134.3	(128.8, 139.7)
Decile 10	9.9	(9.1, 10.9)	134.7	(128.4, 140.9)

Posterior means and 95 % credible intervals

However, the multiple morbidity contrast is greater for females, namely 59 % higher in the most deprived neighbourhoods. Similarly, the correlation between area multiple morbidity rates (posterior means) and IMD scores is higher among females than males, 0.82 as against 0.68. By contrast, the correlation between area mortality rates (posterior means) and IMD scores is higher among males than females, 0.72 as against 0.53.

**Fig. 3:**
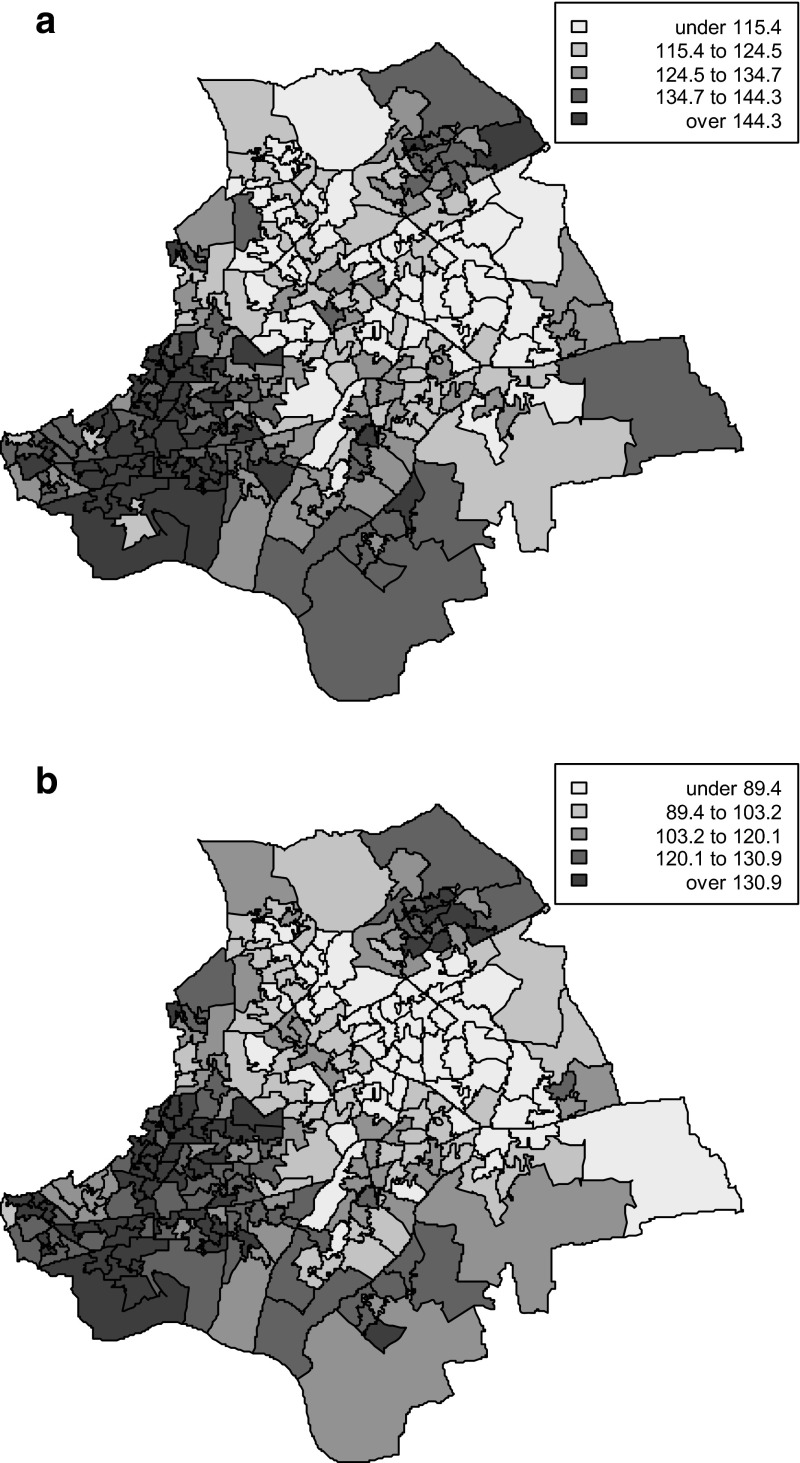
**a** Multi‐morbidity rates (per 1000, all ages) by small area (males). **b** Multi‐morbidity rates (per 1000, all ages) by small area (females)

Figure 3a, b map out posterior mean rates of all age multiple morbidity, R_a_, for each gender. There is significant spatial clustering, with Moran’s I (Tsai 2012) having mean (95 % CRI) of 0.68 (0.63, 0.73) for females, and 0.56 (0.49, 0.62) for males. By comparison, Moran’s I for area mortality rates are much lower, having means (95 % CRI) of 0.34 (0.25, 0.41) for males, and 0.28 (0.21, 0.35) for females.

## Conclusion

As noted by Mercer et al. (2009), multi-morbidity is increasingly the norm in primary care patients and will become more common as populations age. They also note that multi-morbidity is not confined to old age, and studies are needed of multi-morbidity across the life-course.

In this spirit, the present analysis adopts a life table perspective to multi-morbidity while also considering spatial contrasts, especially those related to area socio-economic status. The analysis shows that while rates of multi-morbidity are highest among the very old, spatial contrasts at these ages are relatively small. However, spatial contrasts in multi-morbidity at middle and early old ages (50–74) are considerable.

Such spatial contrasts are closely linked to area deprivation levels, and links between multi-morbidity and deprivation are stronger among females, whereas links between mortality and deprivation are stronger among males. High spatial clustering in multi-morbidity is also evident, reflecting in part that area risk factors such as deprivation are also spatially clustered. Bivariate spatial dependence between health and deprivation is stronger for multiple morbidity than mortality.

Studies of impacts on health care usage of multi-morbid patients indicate they have more contacts with primary care, more prescriptions, more referrals to specialized care (van Oostrom et al. 2014), and higher rates of unplanned hospital admission (Payne et al. 2013). The analysis here has shown differentials in emergency admissions and inpatient bed days to be particularly marked in early old age (65–74). Coupled with evidence of wide geographic contrasts in proportions of early old age spent in multiple morbidity (Table 4; Fig. 1), the implication for geographic variation in health care burdens is clear. This has relevance for area health need indices, often simply based on various measures of socioeconomic status (e.g. Sundquist et al. 2003), or sometimes including rates of long term illness (albeit based on a binary contrast in health status without regard to possible multi-morbidity). The evidence here confirms that differences in multi-morbidity between areas are also potentially important in population risk segmentation, with regard to particular outcomes such as unplanned admissions (Paton et al. 2015).

Thus the present analysis suggests that health need indices should more explicitly consider the structuring of illness patterns, especially the proportion of all patients with multiple chronic disease, and the proportion of people aged 50–74 with multiple conditions. If the preference is for need indices based purely on socioeconomic status, then the present analysis suggests that multiple morbidity be one outcome which is used to validate such indices when used for predicting health needs (Gordon 2003).
